# Limberg’s Rhombic Flap: A Review of Embryological, Anatomical, and Clinical Considerations

**DOI:** 10.7759/cureus.85546

**Published:** 2025-06-08

**Authors:** Maria Paraskevi Lamprou, Dimosthenis Chrysikos, Alexandros Samolis, Maria Piagkou, Dimitrios Filippou, Theodore Troupis

**Affiliations:** 1 Department of Anatomy, National and Kapodistrian University of Athens School of Medicine, Athens, GRC

**Keywords:** anatomy, complications, contraindications, flap design, indications, limberg flap, modifications, rhombic flap, technique

## Abstract

Nearly a century ago, a new surgical approach was introduced for reconstructing rhombus-shaped defects by designing a highly versatile, geometrical, transpositional local flap, combining rotation and lateral shift. Known as the Limberg flap, this unique design is also known as the rhombic flap or, less precisely, the rhomboid flap. The rhombic flap is a full-thickness flap with a random pattern of blood supply, relying on vascularization from the dermal-subdermal plexus. It offers minimal donor site morbidity, reliable neurovascular supply, and a favorable aesthetic outcome. Over time, rhombic flaps have been widely adopted for the closure of small to medium-sized defects resulting from various clinical conditions across different anatomical regions. Successful implementation of this technique requires, first and foremost, a solid understanding of the embryological and anatomical characteristics of the flap, followed by a detailed overview of the exact technique and its modifications, as well as a step-by-step analysis of each surgical stage. This review aims to present the anatomical principles underlying the rhombic flap, highlight key aspects of its surgical application, including design, technique, contraindications, and complications, and underscore its clinical significance in contemporary reconstructive surgery.

## Introduction and background

The evolution of skin flap surgery closely follows advances in anatomical understanding and surgical planning. A key development was the introduction of the angiosome concept, defining tissue regions extending from skin to bone, supplied by a single artery. Further refinement led to the concept of perforasomes: smaller units each supplied by an individual perforator artery. These units are interconnected by direct and indirect linking vessels, which become functional during flap elevation, allowing perfusion to extend beyond a single perforasome [1]. Translating anatomical theory into operative practice, Professor Alexander Alexandrovich Limberg, a Russian maxillofacial surgeon, introduced a geometric, systemic approach to local flap reconstruction. Limberg designed a transpositional flap for closing 60° and 120° rhombus defects, employing a combination of rotation and lateral shift. Commonly referred to in the literature as the Limberg flap, rhombic flap, or sometimes less accurately, the rhomboid flap, this full-thickness, geometric flap follows the random pattern of blood supply and relies on vascularization from the dermal-subdermal plexus. Its viability is influenced by several factors, including the flap characteristics, such as length-to-width ratio, tissue condition and quality, careful design, and technique, as well as the patient’s medical history. Since its introduction, the rhombic flap has been adopted across various surgical specialties for the reconstruction of small to larger-sized defects in diverse anatomical regions and clinical contexts [2,3]. Due to its geometric clarity, reliable perfusion, and low donor site morbidity, it remains a valuable reconstructive option, particularly in cases where primary closure is not feasible. 

Historical background

Dedicating his career to flap design, Limberg introduced the term “opposite triangular flap” for the first time in 1928 [4]. In 1946, he elaborated on this concept, describing its use for closing rhombus-shaped defects [5]. This original Russian monograph was later translated into English as “Design of Local Flaps,” a chapter in Modern Trends in Plastic Surgery, edited by Thomas Gibson [6]. In 1963, Limberg published his major work, *The Planning of Local Plastic Operations on the Body Surface: Theory and Practice*, which proposed a systemic approach for covering larger defects using adjacent triangular flaps. The English translation of this monograph in 1984 by Wolfe [7] rendered the term “opposite triangular flap” as “convergent triangular flap” [8].

## Review

The terminology

Over the past decade, a linguistic dilemma has emerged in the medical literature concerning the terminology of the Limberg flap as either rhombic or rhomboid. Therefore, it is useful to consider the etymological origin and definition of these two terms. The word “rhombus” derives from the ancient Greek verb “ῥέμβω” (rhémbō), which means to turn around, to spin [9], and it was the name for a child’s spinning top toy [2,10]. In Euclidean plane geometry, a rhombus is an equilateral quadrilateral parallelogram [11], which means that it has four sides, and all the sides have the same length. Ιt has no right angles, and the opposite angles are equal [12]. The adjective “rhombic” describes a noun that has the characteristics and shape of a rhombus. 

On the other hand, a “rhomboid” in two-dimensional geometry is defined as a parallelogram with oblique angles and uneven adjacent sides. Unlike a rhombus, in a rhomboid, only the opposite sides are the same length [13]. As an adjective, “rhomboid” means having the characteristics and shape like that of a rhombus, or it is rhombus-like [2,10]. The close relationship between the definitions of rhombic and rhomboid has led to their erroneous conflation. Even though rhombic is accepted as the most precise and correct descriptor for the Limberg flap in world literature, rhomboid is still more commonly adopted by the authors. 

The term “flap” originates from the Dutch word *“flappe,”* meaning something broad, loose, and suspended, attached by one side only. It refers to a unit of soft tissue being transferred from one site to another while maintaining its own blood supply through a pedicle [14], unlike a tissue graft, which is transferred without its own blood supply [15].

Embryology and anatomy 

Embryological Considerations 

The study of skin development allows for a better understanding of the anatomical features and neurovascular patterns that should be considered during flap design. Embryologically, the skin is composed of two layers: a surface layer, the epidermis, derived from the ectoderm; and a deeper layer, the dermis, derived from the mesenchyme. The epidermis begins to develop from the surface ectoderm following the separation of the neural tube from the overlying ectoderm and the completion of neurulation. By two to three weeks, the ectoderm consists of a single basal layer of cuboidal cells lying atop the underlying mesenchyme, separated by a basement membrane. By four to six weeks, the ectoderm proliferates to form a secondary outer layer of simple squamous, non-keratinizing epithelium, known as the periderm or epitrichium, which serves as the primordium of the epidermis. As the periderm undergoes keratinization, it generates the vernix caseosa, a white waxy substance that protects the forming skin from the amniotic fluid and has an antibacterial effect. By the 11th week, the basal layer progresses into the stratum germinativum, which subsequently, by the 20th week, gives rise to the other four strata of the epidermis: the stratum spinosum, stratum granulosum, stratum lucidum (in palms and soles), and stratum corneum [16]. 

During the second month, mesenchymal cells closest to the ectoderm differentiate into dermal cells. The main component of the dermis is collagen, and depending on its texture, two distinct layers of the dermis can be identified: the papillary layer, which extends upward into the epidermis (epidermal-mesenchymal interactions develop the dermal papillae and the dermal ridges and grooves); and the reticular layer underneath, containing a large amount of collagen fibers. Immediately below the dermis lies the hypodermis, or subcutis, which, over most of the body, is characterized by a thick layer of adipose tissue [16,17]. The development of the dermis also includes the neurovascular supply, which starts within the papillae and is complete by the end of the first trimester of gestation. Thus, by the eighth week, the primitive cells are organized parallel to the epidermis, forming the subpapillary plexus. By seven to 10 weeks, a second deeper horizontal plexus appears, and both plexuses extend to acquire the prenatal vascular pattern of arterioles, venules, and capillaries. Only after the first few postnatal weeks is the definitive adult morphology reached, and the final blood supply of the skin arises from musculocutaneous, direct cutaneous, and septocutaneous arteries [16,18]. 

Mechanical forces also play a central role in skin morphogenesis. Tension within the tissue directs the alignment of epidermal keratinocytes and dermal fibroblasts through interactions with extracellular matrix (ECM) fibers and structures such as hair follicles, aligning along axes of mechanical stress, commonly referred to as Langer’s lines. This balance of mechanical forces, known as tensional homeostasis, is essential for maintaining skin structure, fibroblast orientation, collagen fiber organization, and skin contraction [19]. Similarly, during wound healing, fibroblasts interact with the ECM, as collagen fibrils align to generate mechanical tension vectors that guide their migration and differentiation into contractile myofibroblasts. Proper ECM tension supports collagen fiber organization along pre-existing tension lines, thereby facilitating efficient tissue repair. However, excessive tension may lead to hypertrophic scar formation, whereas insufficient tension can impair fibroblast function and collagen production, resulting in suboptimal wound healing outcomes [20]. Understanding the predefined orientation of skin tension lines has direct implications for surgical planning and regenerative outcomes. 

Anatomical Considerations 

The microcirculatory system of the skin is composed of several interconnected vascular plexuses. Five distinct plexuses are described within the skin: the dermal and subdermal (often considered a single unit), the subcutaneous, the prefascial, and the fascial networks. The collateral blood flow that occurs in the skin's vascular anatomy enables a significant degree of vascular redundancy. This redundancy ensures adequate nutrient flow into the flap, promoting its survival and healing [18]. The skin is innervated by both the sensory and sympathetic nervous systems. During flap incision, the sympathetic nerves are transected. As a result, catecholamines are released from nerve terminals, while neurotransmitter reuptake is eliminated, leading to local vasoconstriction. This homeostatic mechanism minimizes blood loss, but also reduces critical blood flow within the flap. However, after one or two days, the loss of sympathetic tone induces vasodilation in the network supplying the flap, enhancing its viability. Neovascularization begins on days three to seven, following transfer via angiogenic and vasculogenic processes [16,18,21]. 

The rhombic flap is a unique, multilayered, local flap due to its geometric transpositional design and random pattern blood supply. This full-thickness flap depends on the dermal-subdermal plexus for vascularization. Structurally, this plexus is found between the hypodermal layer and the reticular layer of the dermis, where numerous arterial anastomoses are present [3]. The preservation of the blood supply and the viability of the flap are influenced by the length-to-width ratio of the pedicle. A length-to-width ratio of 2:1 to 4:1 is generally considered a safe maximum for the head and neck, while a 1:1 ratio should be assessed for areas with insufficient blood supply [21]. Furthermore, the inclusion of a certain amount of subcutaneous fat, which preserves the subdermal plexus in the pedicle, as well as minimal trauma to the pedicle during elevation, will enhance flap viability and lower the risk of necrosis [3]. Although rhombic flaps exhibit a random blood supply, perforator vessels at the base of the flap may need to be identified and preserved in larger defects. Doppler ultrasonography may be used for this purpose [14]. 

Flap design 

Original Limberg Flap 

The Limberg flap was designed for the closure of 60°-120° rhombus defects with a combination of rotation and lateral shift. It represents the transposition of equilateral triangles and can be conceptualized as a variant of the Z-plasty [22]. For a successful flap transfer, especially from areas with relatively lax skin, it is essential to identify and evaluate both the relaxed skin tension lines (RSTLs) and the lines of maximum extensibility (LME) [3]. The RSTLs reflect the natural direction of minimal skin tension in a relaxed state and are more clinically reliable than traditional Langer's lines, which were derived from cadaveric studies. Although in many areas of the body the RSTLs and Langer's lines run in similar directions, in regions with complex movement, such as the corners of the mouth, the temples, the lateral canthal area, and particularly the forehead, they can diverge significantly. Despite their limitations, Langer's lines are still commonly referenced in the literature, often being confused with RSTLs or wrinkle lines, which may lead to clinical misinterpretation [23-25]. 

The RSTLs are not visible like wrinkle lines, but they can be detected in vivo by gently pinching the skin and observing the direction of the ridges and furrows that form. When pinching is done perpendicular to the RSTLs, the skin produces longer, more uniform furrows that align symmetrically on each side of the pinch. If the pinching is done obliquely, the resulting furrows may form an irregular S-shaped pattern. This technique is considered the most accurate for identifying RSTLs, compared to joint mobilization or muscle contraction, which can lead to misleading lines due to temporary changes in skin tension caused by movement. These temporary changes do not reflect the permanent, natural tension of the RSTLs, which are shaped by the underlying bone, cartilage, and soft tissue. The RSTLs exert a constant tension, even during rest or sleep. The LMEs, on the other hand, lie at a 90° angle to RSTLs and indicate the direction in which the skin can stretch more easily. Recognizing these lines is crucial for reconstructive procedures, as designing flaps in alignment with LMEs can optimize mobility, while incisions placed along RSTLs minimize tension and support better wound healing [21,23,25]. 

**Figure 1:**
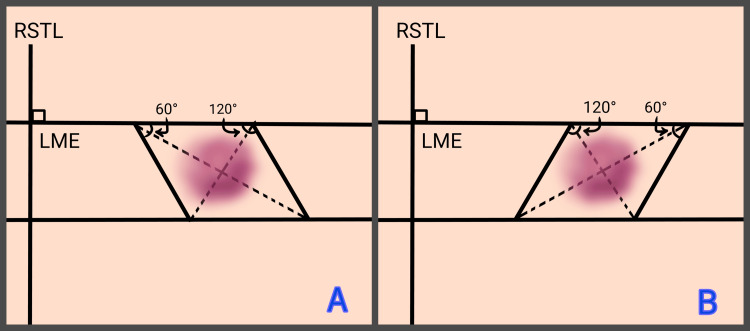
Outline of the defect in a rhombus shape. Two possible rhombi can be drawn. A: Two parallel lines are drawn along the LME axis, enclosing the defect (marked with a purple hue). Two additional lines are drawn medially and laterally to form angles of 60° and 120°. The axis connecting the acute angles (60°) of the rhombus is referred to as the long axis, while the axis between the obtuse angles (120°) is referred to as the short axis.​ B: Rhombus reoriented; same defect enclosed with altered axis alignment. RSTL: Relaxed skin tension lines; LME: Lines of maximum extensibility

**Figure 2:**
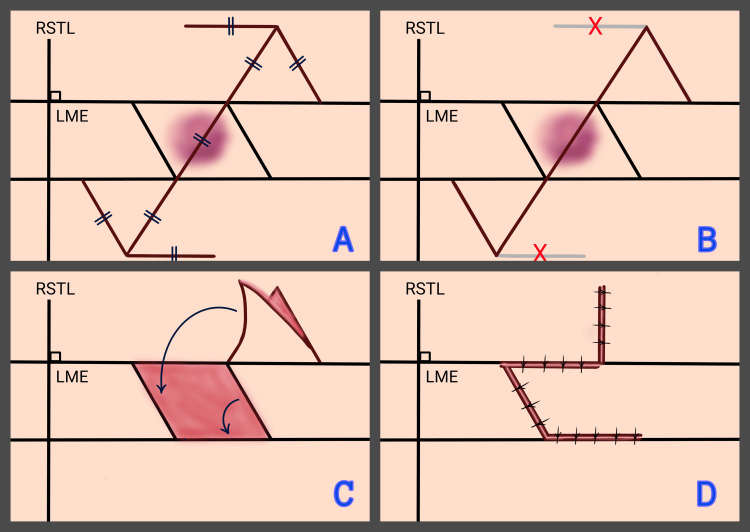
Step-by-step planning and execution of a rhombic flap for skin defect reconstruction​. A: The flap is designed by extending the short axis of the rhombus and drawing the second side at a 60° angle, parallel to the closest side of the rhombus; all sides are equal in length.​ Four flap options exist per rhombus. B: Only two flap options are viable. The ideal flap aligns with the LME and allows closure along the RSTLs​. C: The flap rotates into the primary defect (shown in red in rhombus shape) to restore the area​. D: Closure of the primary defect is achieved, while the secondary defect is closed along the RSTLs. The final result resembles a zigzag line or a question mark. RSTL: Relaxed skin tension lines; LME: Lines of maximum extensibility

The design begins with the outline of the defect in a rhombus shape. Two parallel lines are drawn along the LME axis, enclosing the defect while maintaining sufficient resection margins. Two additional lines are drawn medially and laterally to form angles of 60° and 120°, resulting in the creation of two possible rhombi. The axis connecting the acute angles (60°) of the rhombus is referred to as the “long axis,” while the axis between the obtuse angles (120°) is referred to as the “short axis” (Figures 1A-1B). The first side of the flap is created by extending the short axis of the rhombus from either side by a distance equal to its own length. The second side of the flap is drawn parallel to the side of the rhombus that is closest to the direction of the second side of the flap, at an angle of 60 degrees, and at the same length as earlier [18]. All sides of both the rhombus and the flap are equal in length. Four flaps can be designed for each geometric rhombus (Figure 2A), although only two are typically feasible in clinical practice [2,3,26]. In these cases, the imaginary line forming this equilateral triangle of the flap should extend along the LME, which is perpendicular to the RSTLs (Figure 2B). This orientation allows the flap to rotate into the primary defect while the secondary defect is closed along the RSTLs [14]. The final result resembles the shape of a “zigzag” line or “question mark” (Figures 2C-2D). The final choice of the flap also considers adjacent anatomical landmarks and the characteristics of aesthetic subunits, including skin thickness, pigmentation, and the presence or absence of hair [2,3,27]. 

Modifications 

**Figure 3:**
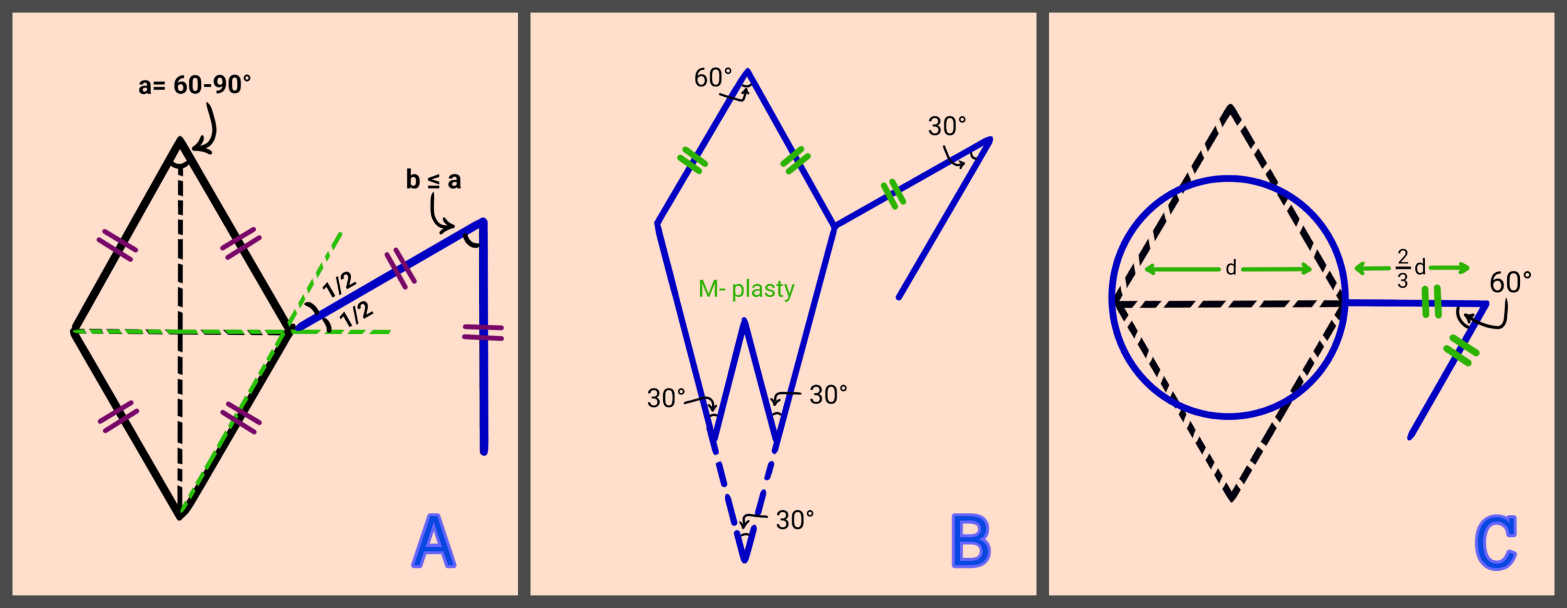
Modifications of the Limberg flap A: The Dufourmentel flap (1962) — The initial incision (shown in blue) bisects the angle formed between two imaginary lines (shown in green), one along the short diagonal and one from an adjacent side of the rhombus. A second incision (shown in blue) is made parallel to the long diagonal of the defect. ​ B: Webster flap (1978) — The 60° base angle of the rhombic defect is divided into two 30° angles using an M-plasty configuration. C: Quaba and Sommerland flap (1987) — A circular defect is reconstructed with a rhombic flap whose sides equal two-thirds of the circle's diameter, maintaining a 60° tip angle.​

In 1962, Claude Dufourmentel modified the Limberg flap design by expanding the acceptable acute angle range of the rhombic defect to 60° to 90° and introducing an alternative flap configuration. The initial incision bisected the angle formed between two imaginary lines: one extending from the short diagonal axis and the other from an adjacent side of the defect. The second incision was aligned parallel to the long diagonal axis. The flap’s sides were equal in length to those of the defect, though not to the short diagonal, and its angle could either match or be smaller than that of the original defect (Figure 3A). Despite the increased geometric complexity, the broader pedicle and more flexible design allowed for improved vascularization, reduced mobilization of healthy tissue, and easier donor site closure [3,28]. 

In 1978, Richard Webster observed that reducing the apical angles of the flap facilitated more straightforward closure of the resulting secondary defects. Accordingly, he designed a 30° angled flap and divided the 60° angle at the base of the rhombic defect using an M-plasty technique, thereby creating two 30° angles (Figure 3B). This approach resulted in reduced donor site tension and a lower risk of standing cone formation or tissue distortion [29]. 

In 1987, Quaba and Sommerland replaced the classic rhombic defect with a circular one, which they reconstructed using a smaller rhombic flap. The sides of the flap were equal to two-thirds of the circle’s diameter, while maintaining the 60° angle characteristic of the Limberg flap (Figure 3C). In addition to incorporating the advantages of previous modifications, the Quaba/Sommerland flap preserved healthy tissue by eliminating the need to convert the circular defect into a rhombic shape [30].

Over the last two decades, different approaches have been published attempting to adjust the classic Limberg flap to various clinical situations, improving its outcomes. Several local Limberg flap modifications have been proposed by Lister and Gibson, and El-Tawil et al., Jervis et al., Karacor-Altuntas et al., and Turan et al., utilizing two [31,32], three [33,34], and four [35] rhombic flaps, respectively, for the closure of larger and more complex defects. Mizutani et al. suggested modified local flaps to minimize the loss of healthy skin during the removal of round tumors in different anatomical regions [36]. More recently, a slightly modified Dufourmental flap has been proposed for a more natural-looking result in skin defect reconstruction [37].

Surgical application

Preoperative Considerations

Assessment of contraindications: Several factors should be assessed preoperatively to determine whether a patient is a potential candidate for the surgical procedure and to detect any reversible factors or contraindications that may increase intraoperative and postoperative complications. Patient factors, including lifestyle, such as nutrition and glucose control, nicotine exposure, and BMI, as well as past medical history and medications, should be documented. Contraindications related to the patient’s health status include uncontrolled diabetes, tobacco use, low BMI (indicating less available skin), vascular diseases, anticoagulation, radiation, and immunosuppressive therapy [14]. However, preoperative lifestyle modifications and medication adjustments may help limit postoperative complications [3]. 

The characteristics of both the defect and donor site need to be thoroughly examined. The size and location of the defect, the available tissue, and its condition and quality [35], along with RSTLs and vector directions, should be investigated and primary and secondary scars should be properly oriented. Anatomical areas with poor surrounding skin laxity, such as the scalp, forehead, including the glabella, brow, and temporal region, the caudal third of the nose, shoulders, skin over extensor joints, palm of the hand, anterior lower extremity, and lower posterior extremity are generally unfavorable for reconstruction [38,39]. In addition, areas with critical anatomical landmarks, such as the eyes, nostrils, oral commissures, and eyebrows [40], and previously reconstructed sites, should be avoided. Inadequate margin clearance in oncological patients is a contraindication to reconstruction, and the operation should be delayed until negative margins are confirmed [2,3]. While large defects have traditionally been considered a contraindication for rhombic flap reconstruction, successful case reports have demonstrated its feasibility [14]. 

Intraoperative Considerations

Technique: The patient is placed in the optimal position for the surgical procedure. After marking the rhombic flap, the appropriate type of anesthesia is administered. Most rhombic flaps can be performed under local or regional anesthesia, though general anesthesia and intravenous sedation may be planned for larger defects, patients with low pain tolerance [2], or those with coexisting factors. When administering local anesthesia, excessive injection of vasoconstrictors, such as epinephrine, around the vascular pedicle and underneath the flap should be avoided to prevent compromising flap perfusion. Injecting the anesthetic five to 10 minutes before making the incision may improve hemostasis [40]. The area is draped in a standard sterile and antiseptic manner [14]. 

The lesion is excised with confirmed clear margins, and the excision is extended to form a rhombus-shaped defect. The two limbs of the flap are then incised and raised with the assistance of different surgical tools, such as dissecting scissors, scalpel blades, and skin hooks. During the flap harvest, it is essential to ensure meticulous hemostasis. Bleeding commonly occurs during the initial incision or while undermining the flap, and careful dissection at a uniform plane and the correct depth minimizes blood loss. Adequate hemostasis and hematoma prevention are achieved through the use of bipolar diathermy during the flap harvest. However, aggressive electrocautery should be avoided, as it may damage the dermal and subdermal plexuses crucial for flap perfusion. For optimal aesthetic results, it is important to ensure that the depth of the donor tissue matches the rhombic defect, and that tissue elevation is performed within the same anatomical plane. The donor tissue is rotated into the defect to evaluate tension and any potential deformity [2,3,40]. 

Closure should start from the donor site, since there is more tension, especially at the apex, and continue with the flap transfer and stabilization into the defect. For safer and more secure coverage, interrupted or mattress sutures, either absorbable or non-absorbable, can be used. When necessary, deep anchor sutures can be placed to offload tension from the superficial layers and maintain proper flap positioning. Throughout the process, gentle tissue handling is crucial to prevent compression of the flap’s corners and blood vessels, ensuring sufficient blood supply [2,3,40]. Eventually, the final scar resembles the shape of a zigzag line [27] or a question mark [2]. 

Postoperative Considerations 

Postoperative management involves health monitoring and support, incision care, and early recognition and treatment of adverse effects that may occur following the operation. For this purpose, close hemodynamic monitoring, nutritional support (including strict glucose control), and psychological interventions may be recommended. Antibiotic ointment may be applied along the sutures, which are removed after one to two weeks, depending on the location [14]. 

Postoperative complications are common, and vigilance for early signs of infection, internal hemorrhage or wound bleeding, pain, and abnormal scarring is required. Flap-related complications, particularly those involving viability, are usually uncommon. Partial or complete flap necrosis has a high incidence among patients with predisposing or contraindicating factors. Flap failure may be prevented through gentle tissue handling and avoidance of intraoperative traumatic techniques. Additionally, excessive traumatic techniques increase the risk of damaging the underlying structure and its components. Aesthetic deformities, such as standing cones ('dog-ear') formation, pincushioning and widened scars, may develop due to tension distribution issues, impaired lymphatic drainage [3], transposition approach, and special anatomic features, such as location, skin elasticity, or thickness [41]. Different aesthetic treatments, like fillers, chemotherapy, laser, and scar modifiers [2], combined with careful preoperative planning and experienced intraoperative technique, may result in a favorable cosmetic outcome [41,42]. It is necessary for patients to be thoroughly informed about all possible complications before consenting to the operation. 

Clinical significance 

The rhombic flap is a simple and effective local flap that provides good cosmetic outcomes. It is designed instantly without the need for special considerations. Thanks to its unique geometry, the flap minimizes donor site morbidity and ensures a reliable neurovascular supply. Given that the neighboring skin is mobilized for the closure of the defect following Gillies’ “like for like” principles, it results in an excellent match of skin color, texture and thickness enhancing patient satisfaction [12,14]. 

A century after it was first introduced by A. A. Limberg, the rhombic flap has been adapted and modified by multiple surgical specialties in a variety of defective locations with different etiologies and sizes, across all demographics, including various age groups, genders, and ethnicities. Rhombic flaps are primarily applied in the head and neck region for facial, central midface, nasal, infraorbital, chin and earlobe defects, dorsally for lumbosacral defects and in the inguinoscrotal and perianal regions. There are also reports of successful use in thoracic, upper and lower limb reconstructions [43-48]. Rhombic flaps have been employed to repair defects caused by skin cancer, lupus, cystic acne, myelomeningoceles, pilonidal sinuses, pressure ulcers, burns, breast cancer, hidradenitis, and life-threatening situations [42-53]. They are particularly indicated for small to medium-sized defects not amenable to primary closure. While traditionally not recommended for larger-sized defects, recent studies have demonstrated the successful application of rhombic flap in excessively large defects [54,55].

## Conclusions

The rhombic flap is a highly versatile and effective reconstructive option, applicable in various anatomical regions and clinical contexts. Successful reconstruction using rhombic flaps requires a solid understanding of skin embryogenesis, cutaneous vascular anatomy, neurovascular supply of the integumentary tissues, and tissue geometry. Meticulous planning and thorough evaluation at every stage of the operation are essential to maximize flap viability and aesthetic outcomes. Preoperative evaluation should always include the examination of the patient and the defect, estimating relative contraindications, and precise surgical planning. During flap transfer, the depth of the tissues, the corners, and the blood vessels should be handled delicately and without force. Postoperative care is crucial to prevent associated complications and adverse effects. The recognition of the unique architecture of the flap and the precise application of surgical principles make the rhombic flap readily applicable in various clinical scenarios, ensuring a successful outcome and an excellent aesthetic result.
